# Recent Progress in Multiple Sclerosis Treatment Using Immune Cells as Targets

**DOI:** 10.3390/pharmaceutics15030728

**Published:** 2023-02-22

**Authors:** Xiaohong Ma, Rong Ma, Mengzhe Zhang, Baicheng Qian, Baoliang Wang, Weijing Yang

**Affiliations:** 1 Department of Neuroscience, The First Affiliated Hospital of Henan University of Chinese Medicine, Zhengzhou 450000, China; 2 The First Clinical Medical School, Henan University of Chinese Medicine, Zhengzhou 450046, China; 3 School of Pharmaceutical Sciences, Henan Key Laboratory of Targeting Therapy and Diagnosis for Critical Diseases, Zhengzhou University, Zhengzhou 450001, China

**Keywords:** multiple sclerosis, immunotherapy, T cells, B cells, innate immune cells

## Abstract

Multiple sclerosis (MS) is an autoimmune-mediated demyelinating disease of the central nervous system. The main pathological features are inflammatory reaction, demyelination, axonal disintegration, reactive gliosis, etc. The etiology and pathogenesis of the disease have not been clarified. The initial studies believed that T cell-mediated cellular immunity is the key to the pathogenesis of MS. In recent years, more and more evidence has shown that B cells and their mediated humoral immune and innate immune cells (such as microglia, dendritic cells, macrophages, etc.) also play an important role in the pathogenesis of MS. This article mainly reviews the research progress of MS by targeting different immune cells and analyzes the action pathways of drugs. The types and mechanisms of immune cells related to the pathogenesis are introduced in detail, and the mechanisms of drugs targeting different immune cells are discussed in depth. This article aims to clarify the pathogenesis and immunotherapy pathway of MS, hoping to find new targets and strategies for the development of therapeutic drugs for MS.

## 1. Introduction

Multiple sclerosis (MS) is an immune-mediated chronic inflammatory demyelination and neurodegeneration of the central nervous system, including immune disorders and imbalances [1]. Globally, it affects about 2 million people, most of them in their 20 s and 40 s, and is the most common non-traumatic disability affecting young people [2,3]. The incidence and prevalence of MS has increased worldwide over the past several decades [4,5,6,7]. The etiology of MS is unknown and may be related to genetic susceptibility, viral infections, environmental factors, vitamin D deficiency, sex differences, and intestinal microbiota dysregulation [8,9,10,11,12,13]. Some of these factors activate the human immune system, causing immune dysregulation, leading to an immune attack against the myelin sheaths of the central nervous system. Immune dysregulation is an important feature of MS, mani.fested by infiltration of immune cells in the central nervous system, triggering myelin depletion, axonal damage, gliosis, and neuronal degeneration [14]. Thus, targeted immune-cell therapy is therapeutically effective [15].

**Figure 1:**
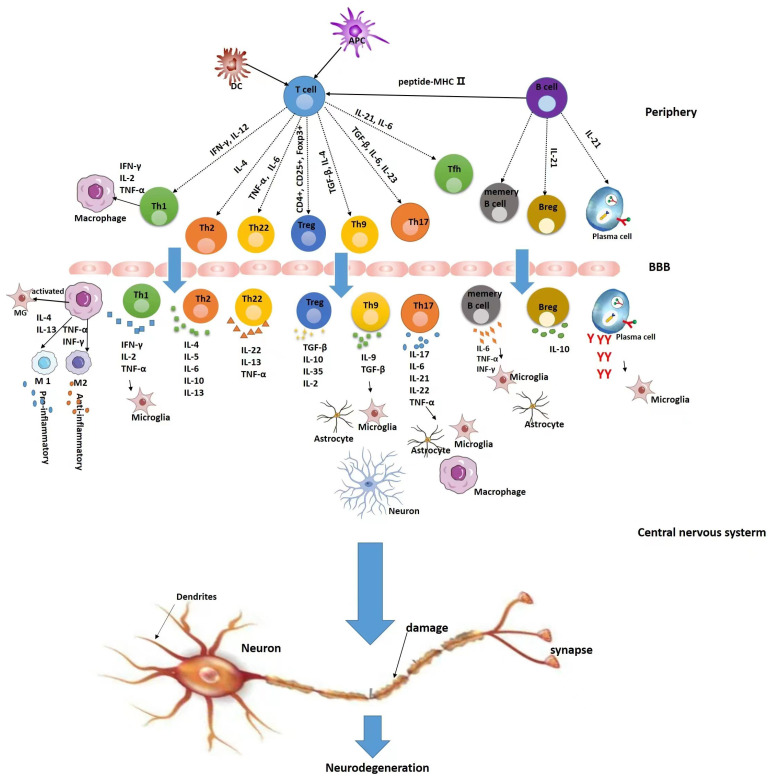
Pathways of action of different immune cells in the pathogenesis of multiple sclerosis.

MS has long been recognized as a classic autoimmune disease mediated by T cells. As the immunopathology of MS becomes more thoroughly investigated, B cells and their mediators of humoral and innate immune cells also play an important role in the pathogenesis of multiple sclerosis [16,17,18,19,20]. Currently, MS remains incurable. Current disease-modifying therapies (DMTs) have made some progress, but they cannot stop the disability accumulation associated with disease progression, and extensively suppress the function of immune effector cells and cause serious side effects [21,22,23]. Thus, therapies based on targeting different immune cells are of growing concern. This article reviews the pathogenesis of immune cell types and mechanisms of action (Figure 1) and explores pharmacological pathways targeting different immune cells as therapeutic targets. It is hoped that new targets and strategies will be sought for the development of pharmaceuticals for multiple sclerosis.

## 2. Intrinsic Immune Cells and MS

Studies have found that innate immune cells, such as microglia, macrophages, and dendritic cells, play important roles in MS pathogenesis. Currently, drugs targeting MS also have regulatory effects on these innate immune cells. An overview of the possible mechanisms of action of innate immune cells in the pathogenesis of MS and the pathways of action of relevant therapeutic agents is now presented (Table 1).

### 2.1. Microglia and Macrophages

Microglia (MG) are resident innate immune cells in the central nervous system (CNS), while macrophages are infiltrating cells [32]. Both cells are phagocytic and participate in CNS immune balance, inflammatory responses, and tissue repair [33,34]. MG was found to overexpress multiple major histocompatibility complex II (MHC II) and co-stimulatory molecules, and to secrete large quantities of inflammatory and neurotoxic mediators, promoting the progression of MS disease in MS patients [35]. Under physiological conditions, the MG is in a resting state. When the CNS is activated by exogenous stimuli, proinflammatory and chemokines are secreted to recruit inflammatory cells such as macrophages and T cells into the CNS. Macrophages also secrete proinflammatory and chemokines, further promoting inflammatory progression and inflammatory cell infiltration. At the same time, activated MG also releases matrix metalloproteinases that disrupt the BBB and facilitate the infiltration of inflammatory cells. However, in the later stages of MS, both MG and macrophages release anti-inflammatory cytokines that inhibit inflammation and promote tissue repair, and this change may be related to differences in cell phenotype and secretion of cytokines. It is also known as M1/M2 polarization [36].

Among the drugs used to treat MS, several have been found to regulate both MG and macrophages. Dimethyl fumarate reduces MG activation induced by lipopolysaccharide (LPS) by reducing TNF-α, IL-1β, IL-6, and nitric oxide synthesis, thereby inhibiting MG-associated inflammatory mediator release and relieving MS symptoms [25]. Gliatrol acetate enhanced the phagocytic capacity of mouse monocytes and microglia and promoted the repair of neurologic injury [37]. IFN-β promotes IL-27 secretion by MG and macrophages, which, in turn, inhibits Th17 differentiation and inflammatory responses in experimental autoimmune encephalomyelitis (EAE) [26,27].

### 2.2. Dendritic Cells

DCs play an important role in maintaining the immune balance in the body [38]. DCs can be classified into conventional (cDC) and plasmacytoid (pDC) according to the differences in the transcription factors expressed by DCs and the growth factor ligands on which they depend. The number of cDCs and pDCs was significantly increased in the cerebrospinal fluid (CSF) of MS patients. In EAE, cDC activates CD4^+^ T cells to differentiate into Th1 and Th17 cells, whereas pDC promotes Treg differentiation. Studies have shown that DC may have opposing pro-inflammatory and anti-inflammatory functions in EAE and MS, and which functions predominate is related to the stage of disease development, the form of disease, and the type of DC [39]. Among the drugs used for the clinical treatment of MS, there are many that have an effect on DC. For example, dimethyl fumarate inhibits the expression of costimulatory molecules and proinflammatory cytokines in DCs, and glatiramer acetate reduces the expression of costimulatory molecules in DCs [29,30,31].

In summary, a variety of existing DMT drugs can act on innate immune cells. In addition to these drugs, there are drugs that can simultaneously act on multiple cells. For example, IFN-β promotes IL-27 secretion by MG and macrophages, thus inhibiting Th17 differentiation in EAE and inhibiting inflammatory response [26,27]. Meanwhile, interferon β can also promote the apoptosis of mature DCs and inhibit the differentiation of T17 cells mediated by DCs [40]. Another example is that Fingolimod can directly regulate MG and macrophage-mediated immune inflammation and promote tissue repair and myelin regeneration. At the same time, Fingolimod can reduce the migration ability of DCs so as to reduce the inflammatory cell infiltration in the CNS of MS patients [24,28,29,41]. These studies suggest that targeting innate immune cells has great potential for MS therapy.

## 3. T Cells and MS

### 3.1. Mechanisms of T Cells in MS

T cells are the most numerous and functionally complex of lymphocytes. Not only do they play important roles in cellular immunity, they also aid humoral immune responses, so T cells play a central role in adaptive immune responses [42]. T cells can be divided into several subgroups according to different classification methods. For example, CD4^+^ T cells and CD8^+^ T cells were classified according to whether or not they express CD4 or CD8; depending on their function, T cells can be divided into helper T cells (Th) and regulatory T cells (Treg) [43,44]. Among them, all helper T cells (Th) express CD4, commonly referred to as CD4^+^ T cells or Th. Antigen-unstimulated naive CD4^+^ T cells were classified as Th0, which regulated by various cytokines and transcription factors, differentiated into CD4^+^ T cell subsets such as Th1, Th2, Th9, Th17, Th22, and follicular helper T cells (Tfh) [45]. A functional subset of T cells has been found to play an important role in the pathogenesis of multiple sclerosis [46]. In this article we summarize the development of distinct T cell subsets and their cytokines in the pathogenesis of MS.

#### 3.1.1. Th1/Th2

CD4^+^ T cells have been found to play an important role in promoting the autoimmune response in MS patients [47]. In 1986, Mosmann et al. proposed that CD4^+^ T cells could differentiate into two functionally opposing subsets, namely, Th1 and Th2 cells [48]. Th1 cells mainly mediate cytotoxicity by secreting Interferon gamma (IFN-γ), TNF-α, and IL -2, etc.; Th1 cells can also activate macrophages to enter the CNS, stimulate oligodendrocytes and cause the destruction of myelin sheaths, thus having a pro-inflammatory effect. Meanwhile, Th1 also inhibits Th2 proliferation. The role of Th2 cells is mainly to produce IL-4, IL-5, IL-6, IL-10, and IL-13 to aid humoral immunity. Furthermore, Th2 has a negative feedback effect on proliferation and differentiation of Th1 cells, is anti-inflammatory, and protects MS [49,50,51]. The autoimmune inflammatory response in MS is associated with an imbalance between Th1/Th2 [52].

#### 3.1.2. Th17/Treg/Th9

Th17 cells are newly discovered Th cells that differ significantly from Th1/Th2 in function and differentiation [53,54]. Th17 mainly secretes IL-17, but also secretes several cytokines including IL-6, IL-21, IL-22, TNF-α, IFN-γ, and granulocyte-macrophage colony stimulating factor (GM-CSF) which are involved in innate immunity and certain types of inflammation, particularly in the development and progression of autoimmune diseases. The murine model of EAE is a classic animal model of MS, commonly used to study the pathogenesis of MS because of its similar pathologic features to MS [55]. The study showed that EAE mice have elevated levels of Th17 cells and IL-17, and that the risk of EAE infection was significantly reduced when Th17 cells were reduced or when IL-6 and IL-23 were lacking [56,57]. Th17 cells have been found to play an important regulatory role in the MS immune response by studying MS patients and EAE [58,59]. Th17 cells activate matrix metalloproteinase expression by inducing secretion of proinflammatory cytokines such as IL-6 and TNF. In addition, it recruits neutrophils to promote inflammation of the CNS, causing inflammatory cell infiltration and disruption of the blood–brain barrier (BBB) [60,61]. Moreover, Th17 activates astrocytes and microglia to form lymphoid-like follicular structures that play important roles in both acute and chronic MS. Cytokines secreted by Th17 play a vital role in the pathogenesis of MS. In a double-blind trial, anti-IL-17 compound reduced lesion formation in MS patients when given to MS patients [62]. In another clinical trial, patients showed deterioration when treated with IFN-γ, so inhibition of IFN-γ production or elimination of the produced IFN-γ could be a treatment for MS [63]. In EAE models, GM-CSF enhanced Th17 differentiation and promoted disease progression, while Th17 cells with GM-CSF deletion completely lost their ability to induce EAE [64,65,66]. Thus, Th17 cells and their secretion are promising new targets for the treatment of MS via the immune pathway.

Treg cells are a subset of CD4^+^ CD25^+^ forkhead box P3^+^ (FOXP3^+^) T cells that regulate a variety of immune functions. Foxp3 is a transcription factor which is not only an important marker of Tregs but is also involved in Treg differentiation. The main function of Tregs is to inhibit the activation of effector T cells and induce immune tolerance, and it can also secrete transforming growth factor beta (TGF-β) and cytokines such as IL-10 to inhibit the immune response and play a negative regulatory role in immunity, thus maintaining the body’s immune balance [67]. Studies have found that in addition to secreting cytokines such as TGF-β and IL-10, Treg cells also secrete IL-35 and IL-2, inhibit T cell proliferation and differentiation, and exert negative immunomodulatory effects [68,69].

The relationship between Th17 and Tregs is close and complex. The differentiation and regulation of Th17 and Tregs are affected by many factors, such as TGF-β, IL-6, aryl hydrocarbon receptor (AHR), and so on. Both Th17 and Tregs are driven by TGF-β [70,71]. IL-6 is a key factor in the differentiation of Th17 and Treg cells, and synergistic interaction of IL-6 with TGF-β inhibits Treg differentiation of CD4+ T cells and promotes their differentiation toward Th17 [72,73]. AHR can regulate the differentiation of Th17 and Treg cells, and AHR agonist can increase the level of Th17 cells and reduce that of Tregs [74]. Th17 and Treg cells have opposing effects: Th17 is pro-inflammatory and induces injury to autoimmune tissues, whereas Treg has anti-inflammatory effects that inhibit autoimmune activation and prevent tissue injury [75,76]. Under normal circumstances, the differentiation of Th17/Treg maintains a dynamic balance that prevents the development of inflammatory diseases [77,78,79]. However, in MS, a Th17/Treg imbalance may be closely related to its pathogenesis. Thus, decreasing Th17 differentiation or promoting Treg proliferation may represent a novel therapeutic strategy for the treatment of MS by targeting immune cells.

Th9 cells are a recently discovered subset of CD4^+^ T cells that play an important role in autoimmune diseases by secreting their signature cytokine IL-9. Th9 differentiates from Th0 cells in the presence of both TGF-β and IL-4 and from Th2 cells induced by TGF-β alone. In addition, IL-9 in combination with TGF-β promotes the differentiation of Th17, which, in turn, promotes the Th17-mediated inflammatory response [80]. On the other hand, IL-9 enhances Treg function and thereby inhibits the immune response [81]. When IL-9 levels were reduced in EAE mice, their symptoms were attenuated, which may be related to the reduction of Th17 and macrophages in the CNS caused by IL-9 depletion [80]. Moreover, IL-9 has been shown to alter the balance of Treg/Th17 cells and to play a dual regulatory role in immune responses [82]. The role of Th9 cells and IL-9 in the pathogenesis of MS is incompletely understood, and there may be two opposing pro- and anti-inflammatory mechanisms regulating its pathogenesis.

#### 3.1.3. Th22

Th22 cells are a novel CD4^+^ T cell. Th22 is differentiated from Th0 cells by TNF-α and IL-6. By secreting IL-22, IL-13, and TNF-α, Th22 participates in epithelial cell physiology and inflammatory pathology, particularly in the immunopathology of inflammatory skin diseases. IL-22, a major cytokine secreted by Th22 cells, is a member of the IL-10 family, which acts primarily on non-immune cells such as epithelial cells and keratinocytes [83,84,85,86]. In addition to being produced by Th22 and Th17 cells, IL-22 can also be secreted by macrophages, natural killer cells, etc. [87]. Recent studies have shown that IL-22 plays an important role in the pathogenesis of autoimmune diseases [88,89,90]. Although the mechanism of action of IL-22 in MS is unknown, there is evidence for an association between the two. Perriard et al. found that serum IL-22 levels were significantly elevated in MS patients and that the expression of IL-22 and its antagonist, IL-22BP (IL-22 binding protein), was dysregulated [91]. Besides, the proportion of Th22 cells and IL-22 in the serum of patients with MS has been found to increase, particularly during disease activity, and is positively associated with disease duration [92,93]. Furthermore, mononucleotide polymorphisms in IL-22R (IL-22RA2) are associated with the pathogenesis of EAE and MS [94,95]. This suggests that Th22 and IL-22 may play important roles in the pathogenesis of MS.

#### 3.1.4. Follicular Helper T Cells

CXC chemokine receptor 5 (CXCR5) is a G protein-coupled receptor for T-lymphocyte migration to the B-cell region of lymph nodes. CXCR5 is mainly expressed on the surface of T lymphocytes, macrophages, and monocytes. The expression of CCXR5 receptor and its ligands Macrophage inflammatory protein-1α (MIP-1a) and MIP-1β is up-regulated in active lesions of MS patients, suggesting that chemokines and their ligands are necessary in the pathogenesis of MS [96]. Tfh cells are a subset of CD4^+^ T cells present in the peripheral immune organ phenotype CD4 CXCR5. The marker cytokine IL-21 produced by Tfh cells plays an important role in B cell differentiation into plasma cells, production of antibodies, and switching of immunoglobulin lineages. In addition, Tfh is an important cell that aids B cell immune responses and maintains T and B cell homeostasis [97,98,99,100]. Circulating memory Tfh cell numbers and plasma IL-21 levels have been found to be significantly increased in relapsing MS patients but decreased in patients in remission. Thus, it has been postulated that circulating memory Tfh cells may be associated with MS recurrence [101]. Studies have found that Tfh cells infiltrated the CNS of EAE mice. As the proportion of Tfh increased, treatment with anti-Tfh chemokine ligands significantly reduced the incidence of EAE disease [102]. The mechanism of involvement of Tfh cells in MS is unknown, but based on the available evidence, it is speculated that Tfh and IL-21 may be important in predicting MS recurrence and prognosis and may be new targets for the treatment of MS.

### 3.2. Drugs Targeting T Cells for the Treatment of MS

Based on the important role of T lymphocytes in the pathogenesis of MS, most currently approved therapies for MS affect T lymphocyte function. We will focus on drugs that have a major effect on T lymphocytes, such as IFN-beta, glatiramer acetate, and the monoclonal antibody natalizumab, which targets T cells (Table 2).

#### 3.2.1. IFN-β

IFN-β is a cytokine that plays a major role in anti-inflammatory and immunomodulatory actions. In 1993, IFN-β was approved by the US Food and Drug Administration (FDA) as a first-line drug for the treatment of MS. IFN-β has multiple pathways of action on the immune system, and T cells are the major site of action. Studies have shown that IFN-β can inhibit the activated proliferation of T cells and alter the distribution of T cells within and beyond the BBB and it also inhibits the production of proinflammatory cytokines (e.g., IFN-γ) and induces the increase in anti-inflammatory cytokines such as IL-10 [103,104]. IFN-β can also prevent the differentiation of inflammatory Th1/Th17 cells and change the phenotype of Th cells from inflammatory Th1 to anti-inflammatory Th2 cells. Reducing the levels of Th17 and IL-17 increased the number of inhibitory Tregs [121,122,123,124]. Studies have shown that IFN-β can significantly improve the clinical symptoms of patients, reduce the annual recurrence rate, and delay the progress of the disease [125,126]. IFN-β, used clinically in the treatment of MS, is classified as IFN-β-1a and IFN-β-1b. IFN-β-1a is most frequently used in the treatment of relapsing–remitting MS at a dose of 22 μg or 44 μg intramuscularly three times a week. In addition to its use in relapsing–remitting syndromes, IFN-β-1b can be used in secondary progressive and clinically isolated syndromes, and the recommended dose is 0.2 mg subcutaneously once daily. Both have been shown to reduce the relapse rate by approximately 30% and to moderately slow disability progression [127,128]. 

#### 3.2.2. Glatiramer Acetate

Glatiramer acetate (GA) is a polypeptide compound. In 1996, it was approved by the Food and Drug Administration as the first-line drug for the treatment of relapsing–remitting MS. GA can induce tolerance of myelin-reactive T cells by competitively binding MHC II to myelin, thereby reducing the attack of immune cells on myelin and achieving neuroprotection [105]. At the same time, GA also induces T cell activation and promotes Th1-Th2 transition to exert immunomodulatory effects [106]. Clinical studies have shown that GA reduces 30-year relapse rates and brain activity in patients with MS and that it is effective in the treatment of clinically isolated syndromes [129,130]. Randomized double-blind controlled trials of GA and IFN-β have shown no difference in efficacy between patients with RRMS and clinically isolated syndromes. Furthermore, the combination of GA and IFN-β was not superior to monotherapy in the treatment of MS [131]. In terms of safety, GA was well tolerated, and the main adverse effects were injection site reactions, such as pain and pruritus, which often resolved spontaneously [129].

#### 3.2.3. Natalizumab

With further investigation of the pathogenesis of MS, immunotherapy for MS has also shifted from extensive immunosuppression to highly specific and targeted therapies. Natalizumab is a humanized antibody that selectively acts on lymphocytes and monocytes and prevents T lymphocytes from crossing the BBB [109,110]. In 2004, the drug was approved by the US FDA for the treatment of RRMS in adults. In 2005, it was discontinued because of its potential to cause fatal progressive multifocal leukoencephalopathy (PML), which occurs in about 4.22 out of 1000 patients [111]. In June 2006, natalizumab was re-approved for MS but required strict compliance with risk management regulations. In a randomized, double-blind trial, natalizumab reduced inflammatory brain injury and relapse in patients with relapsing multiple sclerosis compared with placebo [132]. In another randomized, placebo-controlled trial of relapsing multiple sclerosis, natalizumab reduced the annual relapse rate by 68%. Furthermore, the antibody reduced the accumulation of new or enlarged T2 hyperintense lesions by 83% within 1 year and reduced the risk of disability progression by 42% over 2 years [133]. The results of the above studies indicate that natalizumab is effective in reducing the relapse rate, brain lesion reduction, and disability progression in patients with MS. However, since natalizumab almost completely blocks T cell migration to the CNS, T cells are seriously compromised in their ability to defend the CNS, which may lead to PML. Therefore, the adverse effects of natalizumab are still an important issue that needs to be weighed by both physicians and patients.

#### 3.2.4. Fingolimod

As the first FDA-approved modulator of sphingosine-1-phosphate (S1P) receptor, fingolimod can prevent lymphocytes from entering the CNS, reduce the infiltration of inflammatory T cells in the CNS, and slow down the pathological damage of MS [112,113]. Multiple clinical trials have shown that fingolimod can reduce the annualized recurrence rate (ARR) and reduce the number of new lesions on magnetic resonance imaging (MRI) scans in patients with RRMS [134,135,136]. The common adverse reactions of fingolimod included bradycardia, atrioventricular block, macular edema, elevated liver enzyme levels, and mild hypertension, etc. [134].

#### 3.2.5. Teriflunomide

Teriflunomide is the first oral DMT approved by the FDA for the treatment of RRMS. It mainly inhibits lactate dehydrogenase to prevent the synthesis of pyrimidines in lymphocytes and reduce the proliferation of activated T cells and B cells [137]. The recommended dose of teriflunomide is 7 or 14 mg daily, depending on the severity of the patient’s condition and tolerance. Clinical studies have shown that teriflunomide e can significantly reduce the annual recurrence rate of MS patients compared with placebo, and higher doses (14 mg) of teriflunomide can delay the progression of disability in patients [138,139]. In terms of safety, the common adverse reactions of teriflunomide mainly included transaminase elevation, nausea and vomiting, alopecia, etc. [140].

## 4. B Cells and MS

### 4.1. Mechanism of Action of B Cells in MS

MS has long been recognized as a kind of T lymphocyte-mediated autoimmune disease. However, with the in-depth study of the pathogenesis of MS, it is found that B lymphocytes and their mediated humoral immunity may play a more central role [16,141,142,143]. B-cell therapy has demonstrated excellent clinical efficacy in the treatment of MS and confirms the critical role of B cells in the pathogenesis of MS [144]. B cells mediate CNS injury through a variety of mechanisms, including antigen presentation, release of autoreactive antibodies, secretion of proinflammatory cytokines, and formation of ectopic lymphoid tissue.

#### 4.1.1. Presentation of Ags

As antigen presenting cells (APC), B cells are capable of processing antigen into a peptide that is expressed on the cell surface as an antigen peptide-MHC class II complex and presented to CD4^+^ T cells. Subsequently, activated CD4^+^ T cells promote inflammation and disease activity. Subsequently, the antigenic peptide-MHC class II complex activated CD4^+^ T cells, promoting the formation of inflammation and disease activity. Studies have shown that although B cells lacking MHC II can produce auto-antibodies, they cannot stimulate T cells or induce EAE animal models. Thus, B cells in EAE are MHC class II dependent, not antibody-dependent [145,146]. Like T cells, different B cell types have different functions. Of these, effector B cells (Beff) trigger proinflammatory T cell responses, whereas regulatory B cells (Breg) down-regulate inflammation. B cells act as antigen-presenting cells, and their dual role with T cells contributes to the pathogenesis of MS [79,147]. Memory B cells are more efficient antigen-presenting cells and play an important role in T cell self-proliferation [148].

#### 4.1.2. Antibody Generation

Autoreactive B cells are present in the immune fraction of healthy individuals and are in a state of immune tolerance [149,150]. It has been found that ADP ribosylation factor 6 (ARF6) induces endothelial stromalization of the blood vessels, and opens the vascular barrier [151]. After entering the perivascular space, B lymphocytes predominate in the meninges and perivascular spaces of the CNS in MS patients. Stimulation of B cells with autoantigens in the brain releases IgG and binding to myelin, which enhances microglial activation and promotes the formation of MS lesions. On the other hand, autoreactive B cells in the state of immune tolerance develop autoantibodies when stimulated by peripheral antigens. After the autoantibody enters the CNS via the damaged BBB, it forms an antigenic antibody complex with autoantigens in the brain and is phagocytized by macrophages, resulting in tissue damage [152]. Oligoclonal bands (OCBs) are immunoglobulin G synthesized in the sheath, which could be detected in CSF in approximately 90% of MS patients, suggesting an inflammatory CNS response. Therefore, OCBs are also common diagnostic markers of MS [149,153]. However, the pathogenesis of OCBs in MS remains unclear, and no MS-specific autoantigens have been identified [154].

#### 4.1.3. Secretion of Cytokines

B cells also secrete pro- and anti-inflammatory cytokines in patients with MS, and the imbalance between the two is important in the pathogenesis of MS [149,155,156]. In patients with MS, B cells secrete high levels of proinflammatory cytokines such as IL-6, TNF-α, IFN-γ, lymphotoxin (LT), and GM-CSF, which contribute to the inflammatory response in the MS CNS. Among these, IL-6 secreted by B cells promotes the differentiation of pathogenic Th17 and inhibits Tregs, which plays an important role in regulating the Th17/Treg balance [157,158]. Administration of IL-6 inhibitors to mice effectively reduced the incidence of EAE [72]. Moreover, B cells also secrete anti-inflammatory cytokines such as IL-10, IL-35, and IL-4, which negatively regulate the immune response [159,160,161,162]. IL-10 suppresses antigen delivery by down-regulating the expression of costimulatory factors and MHC class II, thereby inhibiting the proliferation of CD4 T cells and the secretion of proinflammatory cytokines such as Th1 and Th17 [163]. In EAE mice, selective knockdown of IL-10-producing B cells worsened the progression of EAE. In contrast, supplementation with B cells that induced IL-10 secretion in vitro inhibited the pathogenesis of EAE [146,164].

#### 4.1.4. Development of Ectopic Lymphatic Tissue

The BBB separates the CNS from the peripheral nervous system. Under pathological conditions, peripheral B cells can cross the BBB and form follicular-like lymphoid tissue or ectopic lymphoid tissue in the CNS. Ectopic lymphoid tissues (ELT) are found in the soft meninges of MS [165]. Germinal center are present in ELT where activated B cells proliferate and differentiate into memory B cells and plasma cells [158,166]. On the other hand, B cells can stimulate the differentiation of T cells into proinflammatory Th17, thereby promoting the development of chronic inflammation. B cells also participate in the maintenance of these structures by producing cytokines, chemokines, and lymphotoxin signaling [167]. The infiltration of these immune cells may be associated with cerebral cortical myelin depletion and neuronal degeneration and disease progression [146,168]. The distribution of ELTs in the meninges is heterogeneous and is seen mainly in the sulcus folds of the deep brain, especially in the cingulate, frontotemporal, and insular regions [169]. The reduced fluid flowing and slower turnover of CSF in these sulcus areas contributes to the accumulation of autoimmune cells and the initiation of inflammatory responses. The presence of ELTs in the meninges of patients with MS promotes disease progression and accelerates mortality. B cells are a major component of ELTs but are insensitive to treatment with CD20 monoclonal antibodies (mAbs), which may be associated with local secretion of B cell survival factor. Thus, more selective, durable, and reversible B cell-directed MS therapies are needed [170].

### 4.2. Drugs Targeting B Cells for the Treatment of MS

The above studies suggest that B cells play an important role in the pathogenesis of MS. In fact, there has been no breakthrough in the long-term therapy of T cells. Since 2008, rituximab has demonstrated excellent efficacy in the control of MS recurrence and has ushered in a new era of B-cell therapy for the treatment of MS. Among several common targets for B-cell-targeted therapies, the most prominent is CD20. The CD20 molecule, which is distributed mainly from pre-B cells to plasmablasts, kills most pathogenic B cells by CD20 monoclonal antibodies, preserves both progenitor B cells and plasma cells, and preserves immune remodeling and humoral immune function.

In recent years, therapies targeting B cells have mainly been anti-CD20 therapies, ranging from the initial human murine chimeric antibody rituximab (65% human) to ocrelizumab (>90% human), with further developments to the all-human ofatumumab. CD20 mAbs induce B-cell damage mainly through selective binding of B cells expressing CD20 antigen, cytotoxicity, and cytophagocytosis [171]. In addition to CD20 monoclonal antibodies, agents that target B cells include alemtuzumab, a monoclonal antibody to CD52, the sphingosine-1 phosphate receptor modulator fingolimod, and dimethyl fumarate, which effectively reduce the number of circulating memory B cells. Here, we will focus on the CD20 monoclonal antibodies rituximab, ocrelizumab, and ofalolimumab (Table 3).

#### 4.2.1. Rituximab

Rituximab, a chimeric IgG1 antibody administered intravenously to human mice, clears circulating CD20^+^ B cells by strong CDC activity and moderate ADCC and ADCP activity [179,180,181]. CD20^+^ B cells may contribute to the pathogenesis of MS by secreting proinflammatory cytokines and regulating T cell responses [182,183]. Thus, rituximab reduces B-lymphocyte-mediated inflammation and relieves clinical symptoms in MS patients by removing circulating CD20^+^ B cells. In a retrospective cohort study, rituximab-treated patients with MS had lower rates of relapse, disease activity, and annual discontinuation compared with other DMTs [184]. In another retrospective multicenter study, rituximab was also effective in treating patients with refractory RRMS who had persistent clinical and MRI disease activity during treatment with other DMTs [185]. Despite these encouraging results, it can only be administered off-label for MS treatment. On the safety side, patients treated with long-term rituximab may develop hypogammaglobulinemia, leukopenia, increased risk of infection, and infusion reactions [172].

#### 4.2.2. Ocrelizumab

Ocrelizumab is a humanized IgG1 monoclonal antibody that exerts killing effects on target cells through antibody-dependent cell-mediated cytotoxicity (ADCC). In March 2017, the antibody was approved for the treatment of adult RRMS and PPMS and was the first drug to be approved for the treatment of PPMS. In two identical phase III trials (OPERAI and OPERAII), controlling for interferon β-1α, the results showed that the annualized relapse rate (ARR) and the percentage of disability progression were lower in the ocrelizumab group than in the control group in both trials [186]. In another clinical trial, ocrelizumab reduced disability progression in patients with MS compared with placebo and reduced T2 lesion volume on MRI in patients with MS [187]. For safety considerations, adverse events commonly associated with ocrelizumab include infusion reactions and infections such as upper and lower respiratory tract infections and skin infections. Active viral hepatitis B is a contraindication to ocrelizumab, and patients should be screened for hepatitis B virus infection before starting ocrelizumab [174,175].

#### 4.2.3. Ofatumumab

Ofatumumab is a subcutaneous humanized monoclonal IgG1 antibody with a low incidence of antimicrobial antibodies. Unlike rituximab and orexinab, it binds to two specific sites of the CD20 molecule on the extracellular loop. Ofatumumab, which binds to CD20 more closely and more persistently, also clears CD20^+^-expressing B lymphocytes, thereby reducing B-cell-mediated inflammation and relieving clinical symptoms in MS patients. In addition to relieving symptomatic symptoms in patients with MS, Ofatumumab has good effects in reducing the rate of relapse with age and is superior to teriflunomide [188]. Ofatumumab infusion has been approved for the treatment of adult patients with relapsing multiple sclerosis (RMS), including clinically isolated syndromes, relapsing–remitting multiple sclerosis, and active secondary progressive multiple sclerosis. For safety considerations, adverse events common to Ofatumumab include injection site reactions, upper respiratory tract infections, and headache [176].

## 5. Conclusions

MS is an autoimmune disease with complex pathogenesis, and T cells have been proposed to play a central role in its pathogenesis. As the study continues, innate immune cells will also play an important role in the pathogenesis of MS. MG and macrophages secrete pro-inflammatory chemokines, recruit inflammatory cells, and promote inflammation in MS early progression. In the late stage of MS, MG and macrophages can release anti-inflammatory cytokines, inhibit inflammatory responses, and promote tissue repair. Dendritic cells also play two distinct roles in the development of MS disease, pro- and anti-inflammatory, depending on the ligands of the transcription factors expressed and the growth factors dependent. With CD20^+^ monoclonal antibodies targeting B cells, including rituximab, oreglizumab, and alfatotumumab, having a significant effect in clinical therapy, perhaps B cells occupy a more central position. B cells play an important role in the pathogenesis of MS by presenting antigens, releasing autoreactive antibodies, secreting pro-inflammatory cytokines, and forming ectopic lymphoid tissue. Based on the available evidence, the onset of MS is the result of the interaction of multiple immune cells. In addition to immunotherapy targeting T cells and B cells, microglia, macrophages, and dendritic cells are potential therapeutic targets for MS, and their secretion of pro- and anti-inflammatory cytokines may be a potential therapeutic target for MS. Inhibition of target cell activation, secretion of pro-inflammatory cytokines, and attenuation of inflammatory cell infiltration in the central nervous system could serve as new strategies for developing therapeutic agents for MS.

## Figures and Tables

**Table 1:** Disease-modifying drugs for multiple sclerosis that are approved to act on innate immune cells.

Cell Types	Drugs	Mechanism of Action	Refs
Microglia	Fingolimod	Downregulates activated microglial production of pro-inflammatory cytokines as TNF-α, IL-1β, and IL-6; Upregulates microglial production of brain-derived neurotrophic factor and glial cell-derived neurotrophic factor	[24]
	Dimethyl fumarate	Reduce the synthesis of TNF-α, IL-1β, IL-6 and nitric oxide, thereby inhibiting MG-associated inflammatory mediator release	[25]
Macrophages	IFN-β	Promotes IL-27 secretion by Microglia and macrophages, inhibit Th17 cell differentiation and inflammatory response	[26,27]
	Fingolimod	Regulate microglia and macrophage mediated immune inflammation, promote tissue repair and myelin regeneration	[24,28]
Dendritic cells	Fingolimod	Down-regulate the expression of CC chemokine receptor 6, reduces the migration of DCs	[29]
	Dimethyl fumarate	Inhibits the expression of costimulatory molecules and proinflammatory cytokines in DCs	[30]
	Glatiramer acetate	Reduces the expression of costimulatory molecules in DCs	[31]

Abbreviations: IFN-β = interferon beta; TNF-α = tumor necrosis factor-alpha; IL = Interleukin; DCs = dendritic cells.

**Table 2:** Disease-modifying drugs for multiple sclerosis that are approved to act on T lymphocytes.

Therapy	Initial Approval (FDA)	Mechanism of Action on T Lymphocytes	Indication	Route and Frequence of Administration	Common Adverse Events	Refs
IFN-β1a/b	1996 (IFN-β1a) 1993 (IFN-β1b)	Inhibit T cell activation and pro-inflammatory cytokine secretion, promote anti-inflammatory cytokine secretion	RRMS	IFN-β1a: IM injection, 3 times a week IFN-β1b: SC injection, once daily	Pain at the injection site, flu-like symptoms, abnormal liver function and endocrine abnormalities	[103,104]
Glatiramer acetate	1996	Induce T cell immune tolerance and promote Th1 to Th2 transformation	RRMS CIS	SC, 3 times a week	Injection site reactions, such as pain, itching, etc.	[105,106]
Mitoxantrone	2000	Extensively inhibit immune cell proliferation (T cells, B cells, macrophages)	RRMS RRMS SPMS	IV, every 3 months	Dose-related cardiomyopathy, promyelocytic leukemia	[107,108]
Natalizumab	2004	Prevent activated T cells from crossing the BBB	RRMS	IV, every 4 weeks	Fatigue and allergic reaction	[109,110,111]
Fingolimod	2010	Sphingosine-1-phosphate Inhibitor, Promote the migration of lymphocytes back to the lymph nodes	RRMS	Oral, once daily	Bradycardia, atrioventricular conduction block, macular edema, elevated liver-enzyme levels, and mild hypertension	[112,113]
Teriflunomide	2012	Inhibit dihydrolactate dehydrogenase, prevent pyrimidine synthesis in lymphocytes, and reduce the proliferation of activated T cells and B cells	RRMS	Oral, once daily	Abnormal liver function (elevated transaminase), gastrointestinal reactions (nausea, diarrhea), hair loss, etc.	[114,115]
Dimethyl fumarate	2013	Inhibit the activation and proliferation of T cells, selectively reduce CD8+ T cells and inflammatory memory subtypes of T and B cells, increasing Treg and Breg	RRMS	Oral, twice daily	Flushing, diarrhea, nausea, upper abdominal pain, Itching, rash, decreased lymphocyte counts, and elevated liver aminotransferase	[116]
Alemtuzumab	2014	Deplete CD52-expressing T cells	RRMS	IV, once daily	Headache, rash, nausea, and pyrexia	[117,118]
Cladribine	2019	Reduce the count of T cells	RRMS SPMS	Oral, 4-5 days over 2-week treatment courses	lymphocytopenia, Headache, nasopharyngitis, upper respiratory tract infection	[119,120]

Abbreviations: IFN-β1a = interferon beta 1a; IFN-β1b = interferon beta 1b; FDA = Food and Drug Administration; RRMS = relapsing–remitting multiple sclerosis; PPMS = primary progressive multiple sclerosis; SPMS = secondary progressive multiple sclerosis; CIS = clinically isolated syndrome; IM = intramuscular; IV = intravenous; IV = intravenous; SC = subcutaneous.

**Table 3:** Monoclonal antibodies that target B cell.

Therapy	Species Isotype	Target	Common Adverse Events	Refs
Rituximab	Chimeric(murine/human)monoclonal IgG1	CD20	Hypogammaglobulinemia, leukopenia, increased risk of infection, and infusion reactions	[172,173]
Ocrelizumab	Humanized monoclonal IgG1	CD20	Infusion reactions and infections such as upper and lower respiratory tract infections and skin infections	[174,175]
Ofatumumab	Humanized monoclonal IgG1	CD20	Injection site reaction, upper respiratory tract infection, headache	[176,177]
Alemtuzumab	Humanized monoclonal IgG1	CD52	Headache, rash, nausea, and pyrexia	[118,178]

## Data Availability

Not applicable.
